# In Cellulo Protein-mRNA Interaction Assay to Determine the Action of G-Quadruplex-Binding Molecules

**DOI:** 10.3390/molecules23123124

**Published:** 2018-11-29

**Authors:** Rodrigo Prado Martins, Sarah Findakly, Chrysoula Daskalogianni, Marie-Paule Teulade-Fichou, Marc Blondel, Robin Fåhraeus

**Affiliations:** 1 Université Paris 7, Inserm, UMR 1162, 75013 Paris, France; rodrigo.prado-martins@inserm.fr (R.P.M.); sarah.findakly@inserm.fr (S.F.); chrysoula.daskalogianni@inserm.fr (C.D.); 2 ICCVS, University of Gdańsk, Science, ul. Wita Stwosza 63, 80-308 Gdańsk, Poland; 3 Chemistry, Modelling and Imaging for Biology, CNRS UMR9187-Inserm U1196, Institut Curie, Université Paris-Sud, F-91405, Orsay, France; mp.teulade-fichou@curie.fr; 4 GGB, Université de Brest, Inserm, CHRU Brest, EFS, UMR 1078, F-29200 Brest, France; 5 Department of Medical Biosciences, Umeå University, 90187 Umeå, Sweden; 6 RECAMO, Masaryk Memorial Cancer Institute, Zluty kopec 7, 65653 Brno, Czech Republic

**Keywords:** structure-activity relationship, protein-mRNA interactions, G-quadruplexes, PhenDC3, pyridostatin, EBNA1, Epstein-Barr virus (EBV)

## Abstract

Protein-RNA interactions (PRIs) control pivotal steps in RNA biogenesis, regulate multiple physiological and pathological cellular networks, and are emerging as important drug targets. However, targeting of specific protein-RNA interactions for therapeutic developments is still poorly advanced. Studies and manipulation of these interactions are technically challenging and in vitro drug screening assays are often hampered due to the complexity of RNA structures. The binding of nucleolin (NCL) to a G-quadruplex (G4) structure in the messenger RNA (mRNA) of the Epstein-Barr virus (EBV)-encoded EBNA1 has emerged as an interesting therapeutic target to interfere with immune evasion of EBV-associated cancers. Using the NCL-EBNA1 mRNA interaction as a model, we describe a quantitative proximity ligation assay (PLA)-based in cellulo approach to determine the structure activity relationship of small chemical G4 ligands. Our results show how different G4 ligands have different effects on NCL binding to G4 of the *EBNA1* mRNA and highlight the importance of in-cellulo screening assays for targeting RNA structure-dependent interactions.

## 1. Introduction

Accumulating evidence indicates an ever-expanding role for RNAs in regulating most aspects of cell biology that range from small non-coding RNAs to messenger RNAs (mRNAs). The more traditional role of mRNAs as “only messengers” is changing and new knowledge is emerging showing how the encoding sequences are taking on more diverse functions and can influence the activity of the encoded proteins. Most, if not all aspects of mRNAs, and this is presumably true also for non-coding RNAs, are governed by interactions with cellular proteins. These ribonucleoproteins (RNP) complexes control RNA metabolism and form scaffolds to orchestrate and organize protein networks and complex functional units [1]. Thus, interfering with specific protein-RNA complexes holds promise for new therapeutic developments as well as furthering our understanding of cell biological process. However, it is challenging to specifically target protein-RNA interactions (PRIs) for several reasons. One reason is that the interactions between proteins and RNAs, in particular mRNAs, are often dependent on RNA structures. However, the folding of RNAs, the chaperones involved, and how RNA-binding protein (RBP) specifically recognize certain RNA structures are still relatively unknown. New evidence that follows technical developments together with studies on disease-related single synonymous mutations have highlighted that folding of RNAs into 3D structures that serve as a protein-binding platform is a regulated and dynamic process encompassing relatively large sequences [2,3,4,5,6]. Thus, RNA structures from the same RNA sequence can be different in vitro as compared to in vivo, making it difficult to set up in vitro-based drug screening assays to identify compounds that interfere with specific PRIs. 

**Figure 1:**
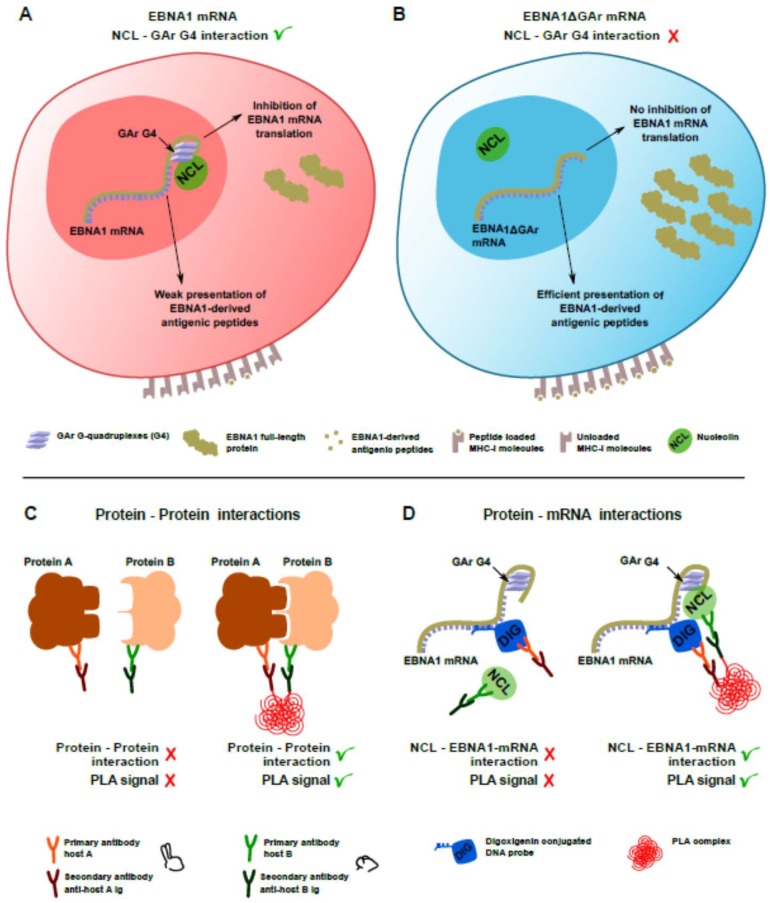
(**A**) cartoon depicting the interaction between nucleolin (NCL) and the G4 formed in the GAr-encoding sequence of *EBNA1* mRNA. This interaction is crucial for EBNA1/Epstein-Barr virus (EBV) immune evasion as it inhibits both *EBNA1* mRNA translation and the production of EBNA1-derived antigenic peptides, hence limiting the production of EBNA1 to the minimal level to fulfill its essential role in maintenance and replication of the viral genome and, at the same time, allowing EBNA1 to evade the immune system. (**B**) If the interaction between EBNA1 G4 and NCL is compromised or lost (e.g., when EBNA1ΔGAr, a form of EBNA1 deleted for its GAr domain, is expressed), then full length EBNA1ΔGAr protein as well as EBNA1-derived antigenic peptides are expressed at a higher level, leading to recognition of EBV-infected cells by the immune system of the host. (**C**) Schematic depicting the principle of the proximity ligation assay (PLA) between two proteins (or two epitopes of the same protein). (**D**) Schematic depicting the adaptation of the PLA to visualize protein/mRNA interactions in cellulo.

G-quadruplexes (G4) are non-canonical nucleic acid structures based on the stacking of several G-quartets, further stabilized by cations positioned in the central channel of the G4 helix [7,8]. These structures are frequently found in eukaryotic transcripts [8] and their formation has been implicated in several steps of gene expression. Indeed, a relevant number of disease-related genes have been shown to be regulated by G4 structures and their RBP [9]. We have recently reported that nucleolin (NCL) directly binds G4 formed in the GAr-encoding sequence of the Epstein Barr virus (EBV) *EBNA1* mRNA (Figure 1A,B, see also Reference [10]). This interaction is critical for minimizing *EBNA1* mRNA translation and thereby the production of EBNA1-derived antigenic peptides for the major histocompatibility (MHC) class I pathway, allowing EBV-infected cells to evade the immune system [11]. As EBV is associated with several human cancers and as all EBV-infected cells express EBNA1, the *EBNA*1 mRNA-NCL interaction represents an interesting target for developing drugs that aim to induce an immune response against EBV-related diseases. It also serves as a broader model for developing techniques required to study structured RNA-protein interactions. Here, we use the NCL*-EBNA1* mRNA interaction to illustrate how different compounds binding to the same G4 have specific effects on the interaction with NCL in cellulo. This illustrates a so far unknown role of G4 structures in mediating specific interactions with proteins, indicating that particular G4-protein interactions can be targeted specifically. We also report how to generate quantitative data using proximity ligation to verify the capacity of different G4 ligands to prevent NCL-*EBNA1* mRNA interactions and their role in controlling *EBNA1* mRNA translation.

## 2. Materials and Methods

### 2.1. Cell Culture, Transfection, and Drug Treatments

The human lung carcinoma cell line H1299 and the EBV-producing marmoset B-cell line B95-8 were cultured in RPMI-1640 supplemented with 10% fetal bovine serum (FBS), 2 mM l-glutamine, 100 U/mL penicillin, and 100 µg/mL streptomycin. H1299 transient transfections were performed using Genejuice reagent (Merck Bioscience, Darmstadt, Germany) according to manufacturer’s protocol. All cells were cultured at 37 °C with 5% CO_2_. For drug treatments, 10^5^ B95-8 cells were incubated with 5 µM of PhenDC3 [12] or pyridostatin (PDS, Sigma-Aldrich (now Merck), Darmstadt, Germany) for 36 h. Drug stock solutions were prepared in DMSO (Euromedex, Strasbourg, France). 

### 2.2. RNA In Situ Hybridization-Immunofluorescence (rISH-IF) and Immunofluorescence (IF)

H1299 cells were plated on 12-mm-diameter coverslips in 24-well plates and transfected with 200 ng of EBNA1 construct [13]. At 24 h post-transfection, cells were fixed with PBS 4% paraformaldehyde for 20 min and then washed with PBS for 10 min. For rISH-IF, samples were overnight incubated in 70% (*v*/*v*) ethanol at 4 °C. After rehydration in PBS for 30 min, samples were permeabilized with PBS 0.4% Triton X-100, 0.05% CHAPS for 10 min at room temperature, and pre-treated with hybridization buffer (10% formamide, 2X SSC, 0.2 mg/mL *E. coli* 522 tRNAs, 0.2 mg/mL sheared salmon sperm DNA and 2 mg/mL BSA) for 30 min at room temperature. Samples were then incubated overnight with 50 ng of an EBNA1-digoxigenin DNA probe (5′ CTTTCCAAACCACCCTCCTTTTTTGCGCCTGCCTCCATCAAAAA-digoxigenin 3′) in a humidified chamber at 37 °C. To avoid secondary structures, the probe was diluted in 5 µL of water, denaturated at 80 °C for 5 min, chilled on ice for 5 min, and resuspended in 35 µL of hybridization buffer. After hybridization, samples were serially washed for 20 min with 2X SSC 10% formamide, hybridization buffer (twice), 2X SSC, and PBS. Washes were carried out at room temperature, except with hybridization buffer (37 °C). Samples were saturated with PBS 3% BSA for 30 min and incubated with a mouse anti-digoxigenin (1/200, clone DI-22, Sigma) for 2 h at room temperature. A goat anti-mouse immunoglobulin G (IgG) secondary antibody conjugated to Alexa Fluor^®^ 568 (Sigma) was used to detect immunocomplexes (1 h at 37 °C) and DAPI was used for nuclear counterstaining under standard conditions. For IF, fixed samples were saturated with PBS 3% BSA for 30 min, incubated with rabbit polyclonal antibody anti-NCL (1/1000, ab22758-Abcam) for 2 h and goat anti-rabbit Ig secondary antibody conjugated to Alexa Fluor^®^ 488 (Sigma) for 1 h at 37 °C. DAPI was used for nuclear counterstaining.

### 2.3. Sequence of EBNA1 cDNA

The sequence of EBNA1 cDNA (GenBank: MG021311.1) is as follows (the GAr-encoding sequence which forms G4 is highlighted in cyan):

ATGTCTGACGAGGGACCAGGTACAGGACCTGGAAATGGCCTAGGACAGAAGGAAGACACATCTGGACCAGACGGCTCCAGCGGCAGTGGACCTCAAAGAAGAGGGGGGGATAACCATGGACGAGGACGGGGAAGAGGACGAGGACGAGGAGGCGGAAGACCAGGAGCTCCGGGCGGCTCAGGATCAGGGCCAAGACATAGAGATGGTGTCCGGAGACCCCAAAAACGTCCAAGTTGCATTGGCTGCAAAGGGGCCCACGGTGGAACAGGAGCAGGAGGAGGGGCAGGAGCAGGAGGGGCAGGAGCAGGAGGAGGGGCAGGAGCAGGAGGAGGGGCAGGAGCAGGAGGAGCAGGAGGAGGGGCAGGAGCAGGAGGAGGGGCAGGAGCAGGAGGAGGGGCAGGAGCAGGAGGAGGGGCAGGAGCAGGAGGAGGGGCAGGAGCAGGAGGAGGGGCAGGAGGAGGAGGAGGGGCAGGAGCAGGAGGAGGGGCAGGAGCAGGAGGAGGGGCAGGAGCAGGAGGAGGGGCAGGAGGGGCAGGAGCAGGAGGAGGGGCAGGAGCAGGAGGAGGGGCAGGAGCAGGAGGAGGGGCAGGAGCAGGAGGGGCAGGAGCAGGAGGAGGGGCAGGAGCAGGAGGGGCAGGAGCAGGAGGAGGGGCAGGAGCAGGAGGAGGGGCAGGAGCAGGAGGGGCAGGAGCAGGAGGGGCAGGAGCAGGAGGGGCAGGAGCAGGAGGGGCAGGAGGAGGAGGAGCAGGAGGGGCAGGAGGGGCAGGAGCAGGAGGGGCAGGAGGGGCAGGAGCAGGAGGAGGGGCAGGAGGGGCAGGAGCAGGAGGAGGGGCAGGAGGGGCAGGAGCAGGAGGGGCAGGAGGGGCAGGAGCAGGAGGGGCAGGAGGGGCAGGAGCAGGAGGGGCAGGAGGGGCAGGAGCAGGAGGAGGGGCAGGAGCAGGAGGGGCAGGAGCAGGAGGTGGAGGCCGGGGTCGAGGAGGCAGTGGAGGCCGGGGTCGAGGAGGTAGTGGAGGCCGGGGTCGAGGAGGTAGTGGAGGCCGCCGGGGTAGAGGACGTGAAAGAGCCAGGGGGGGAAGTCGTGAAAGAGCCAGGGGGAGAGGTCGTGGACGTGGTGAAAAGAGGCCCAGGAGTCCCAGTAGTCAGTCATCATCATCCGGGTCTCCACCGCGCAGGCCCCCTCCAGGTAGAAGGCCATTTTTCCACCCTGTAGGGGAAGCCGATTATTTTGAATACCACCAAGAAGGTGGCCCAGATGGTGAGCCTGACATGCCCCCGGGAGCGATAGAGCAGGGCCCCGCAGATGACCCAGGAGAAGGCCCAAGCACTGGACCCCGGGGTCAGGGTGATGGAGGCAGGCGCAAAAAAGGAGGGTGGTTTGGAAAGCATCGTGGTCAAGGAGGTTCCAACCAGAAATTTGAGAACATTGCAGAAGGTTTAAGAACTCTCCTGGCTAGGTGTCACGTAGAAAGGACTACCGATGAAGGAACTTGGGTCGCCGGTGTGTTCGTATATGGAGGTAGTAAGACCTCCCTTTACAACCTCAGGCGAGGAATTGCCCTTGCTATTCCACAATGTCGTCTTACACCATTGAGTCGTCTCCCCTTTGGAATGGCCCCTGGACCCGGCCCACAACCTGGCCCACTAAGGGAGTCCATTGTCTGTTATTTCATTGTCTTTTTACAAACTCATATATTTGCTGAGGGTTTGAAGGATGCGATTAAGGACCTTGTTATGCCAAAGCCCGCTCCTACCTGCAATATCAAGGCGACTGTGTGCAGCTTTGACGATGGAGTAGATTTGCCTCCCTGGTTTCCACCTATGGTGGAAGGGGCTGCCGCGGAGGGTGATGACGGAGATGACGGAGATGAAGGAGGTGATGGAGATGAGGGTGAGGAAGGGCAGGAGTGA

### 2.4. Proximity Ligation Assay (PLA)

H1299 cells were plated and fixed as previously described. For experiments using B95-8, 12-mm-diameter coverslips were coated with poly-l-lysine 0.01% (Sigma) for 30 min and air-dried for 5 min in 24-well plates. B-cells were then resuspended in PBS, plated on pre-treated coverslips and incubated for 2 h at room temperature. After a wash with PBS for 5 min, cells were fixed with PBS 4% paraformaldehyde for 20 min and re-washed with PBS for 10 min. Following fixation, both cell lines were processed for in situ hybridization according to the rISH-IF protocol. Samples were then saturated with PBS 3% BSA for 30 min and incubated for 2 h at room temperature with a mix of primary antibody containing the mouse anti-digoxigenin and the rabbit anti-nucleolin previously described. Subsequently, PLA was carried out using the Duolink PLA in situ kit (Sigma), anti-rabbit plus and anti-mouse minus probes (Sigma) following the manufacturer’s protocol. 

### 2.5. Microscopy and Image Analysis 

Samples were examined with an LSM 800 confocal laser microscope (Carl Zeiss MicroImaging, Jena, Germany). ImageJ [14] was used for analysis of images. PLA signals were quantified in 100 cells per sample according to the protocol provided as Supplementary materials. Data analysis was carried out by ANOVA with Tukey HSD test using GraphPad Prism 5 for Windows (GraphPad Software, San Diego, CA, USA). Data shown are mean ± SD of three independent experiments.

## 3. Results and Discussion

### 3.1. Detection of EBNA1 mRNA and NCL by rISH-IF and IF

EBNA1 is essential for EBV genome maintenance and, as such, is expressed in all EBV-carrying cells, including cancers. Because EBNA1 is highly antigenic, EBV has evolved a sophisticated strategy to evade the immune detection of latently infected cells by limiting *EBNA1* mRNA translation and, as a consequence, by minimizing the production of antigenic peptides for the major histocompatibility (MHC) class I pathway [13]. Nucleolin (NCL) directly binds G4 structures in the GAr (the glycine-alanine rich domain of EBNA1)-coding sequence of the *EBNA1* mRNA and this is essential for suppressing the translation of the *EBNA1* mRNA and the production of antigenic peptides (Figure 1A,B). A small G4 ligand, PhenDC3, can displace NCL from the *EBNA1* message and augment its translation, thereby increasing the production of antigenic peptides and triggering CD8^+^ T cell response [11]. Interestingly, another study based on in vitro translation showed that pyridostatin (PDS), another G4-binding compound frequently used as benchmark, instead further suppressed *EBNA1* mRNA translation [15]. This observation is surprising, as it has previously not been appreciated that G4 ligands can have different effects on the function of the G4 or on their interaction with cellular factors. In this context, G4 structures might be targeted in a specific way by therapeutic intervention, opening a so far unknown field of drug target validation in cellulo. In order to shed light on the apparent discrepancy between how different G4 ligands act on the NCL-*EBNA1* mRNA interaction and thus their potential use as compounds that can trigger an immune reaction against EBV-carrying cancers, we developed a protocol based on proximity ligation (Figure 1C,D). This approach allows us to overcome difficulties with RNA folding in vitro to quantitatively study endogenous protein-mRNA interactions in cellulo and to avoid using large tags and overexpression system. By combining in-cellulo data with in vitro assays, this approach has the advantage of providing a straightforward and easy confirmation of the relevance of in vitro studies based on recombinant proteins and synthetic RNAs with in vivo data. In addition, we developed a method to quantify protein-mRNA interactions, which could allow structure-activity relationship (SAR) studies in cellulo to be carried out on a specific RNA-protein interaction. 

**Figure 2:**
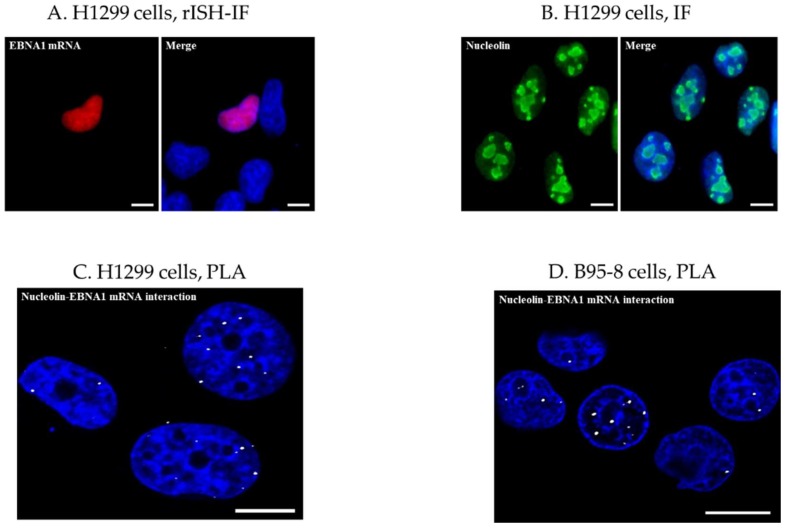
Use of PLA for the study of a protein-mRNA interaction. (A) H1299 cells were transfected with *EBNA1* and analyzed by RNA in situ hybridization-immunofluorescence (rISH-IF) to verify the specificity of the *EBNA1*-digoxigenin probe and to validate the detection of probe-mRNA complexes using a mouse anti-digoxigenin antibody. Immunocomplexes were detected using an anti-mouse Alexa Fluor^®^ 568-conjugated secondary antibody, revealing the accumulation of *EBNA1* mRNA in the nucleus. *EBNA1* mRNA is depicted in red. (**B**) Immunofluorescence (IF) was performed in H1299 cells using a rabbit anti-nucleolin antibody to set up the appropriate conditions for detection of endogenous NCL. The expected labelling of NCL in the nucleolus was confirmed. (**C**) PLA in *EBNA1*-transfected H1299 cells using mouse anti-digoxigenin and rabbit anti-nucleolin tested in (A) and (B). Anti-rabbit and anti-mouse Ig PLA probes were used following the manufacturer’s protocol to generate PLA complexes depicted as white dots. Each dot represents an interaction between NCL and *EBNA1* mRNA. (**D**) The EBV-transformed B-cell line B95-8 was tested for endogenous NCL-*EBNA1* mRNA interaction under the same conditions used in (**C**). PLA uncovered this interaction in the nuclear compartment as in *EBNA1*-transfected H1299 cells shown in (**C**). Scale bars represent 10 µm.

The proximity ligation assay (PLA) was originally conceived to detect proteins in close proximity. For this, one pair of primary antibodies raised in two different species is used to target the two proteins of interest (Figure 1C). Afterwards, DNA strand-conjugated secondary antibodies and enzymatic reactions are employed to generate fluorescent reporter molecules [16]. Since we aimed to explore a protein-mRNA interaction, a hybridization step was added in order to tag the mRNA of interest with a complex recognizable by antibodies (Figure 1D). A similar approach has been reported for DNA-protein interactions [17]. Therefore, as an initial step, EBV negative cells transfected, or not, with an EBNA1 construct were analyzed by RNA in situ hybridization coupled to immunofluorescence (rISH-IF). This enabled us to first validate the hybridization conditions using the digoxigenin-labelled EBNA1 probe and the mouse anti-digoxigenin primary antibody to be employed in the PLA (Figure 2A). IF was also performed to determine the appropriate conditions for detecting endogenous NCL using a rabbit anti-NCL antibody (Figure 2B). Altogether, these assays demonstrated an accumulation of NCL and *EBNA1* mRNA in the nucleus.

### 3.2. Analysis of NCL-EBNA1 mRNA Interactions by Proximity Ligation

PLA was then carried out to detect the NCL-*EBNA1* mRNA interactions in situ using either transfected cells or EBV-infected cells. PLA complexes are depicted as white dots and each dot represents an interaction between NCL and *EBNA1* mRNA. In line with rISH-IF and IF results, interactions were uncovered in or at the close vicinity of the nuclear compartment of EBNA1-transfected cells (Figure 2C) and EBV-transformed lymphoblastic B95-8 cells (Figure 2D), confirming that NCL binds EBNA1 mRNA in the nucleus of virus-carrying cells. During and after transcription, mRNAs undergo a complex maturation process relying on a large repertoire of RBP and indeed, protein-mRNA interactions control pivotal steps of mRNA biogenesis and function [18]. In addition, evidence is accumulating that these interactions play a broader role in cellular processes, regulating multiple enzymatic and metabolic activities [19]. Hence, these results denote that EBV exploits host cell mRNA maturation process/es in order to hamper translation and thus immune responses.

### 3.3. Use of Quantitative PLA to Evaluate the Effect of RNA-Binding Drugs on NCL-EBNA1 mRNA Interactions

**Figure 3:**
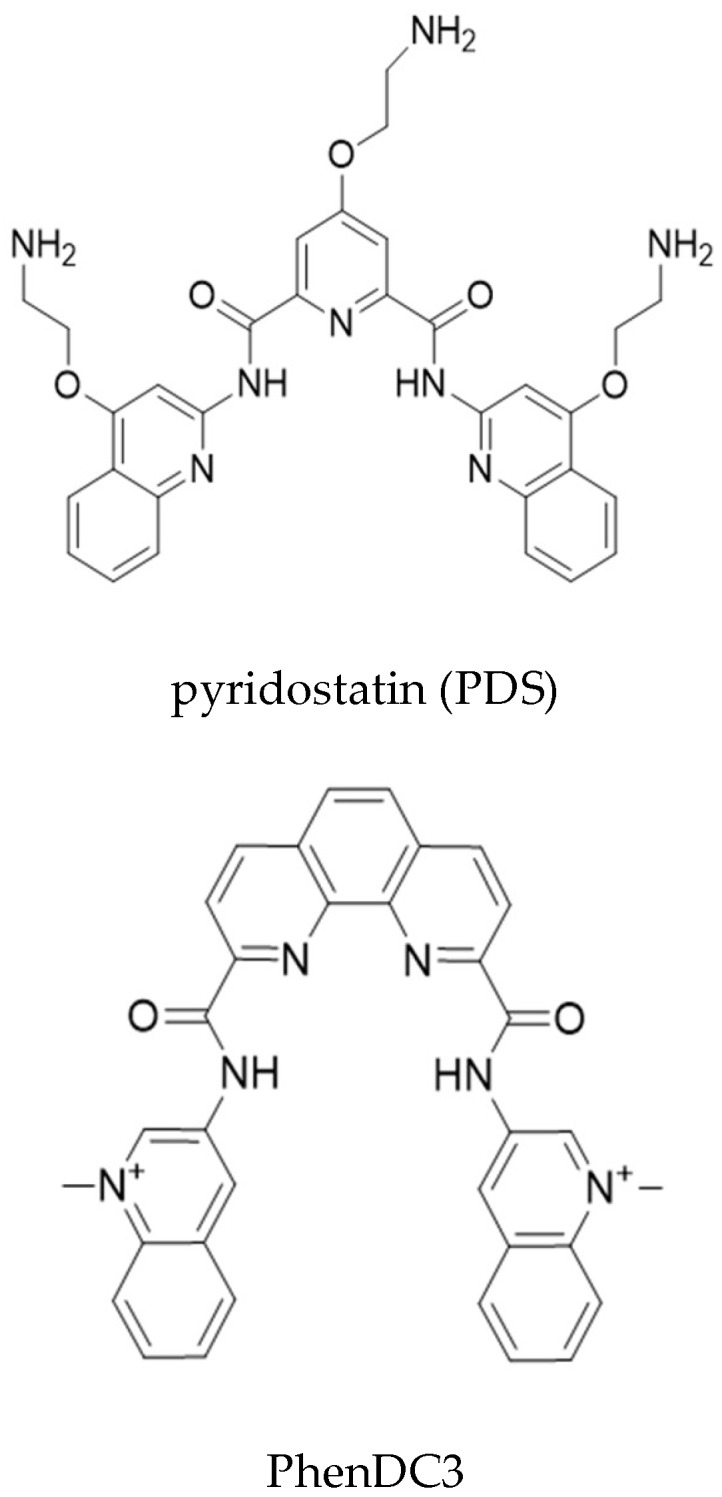
Molecular structures of the G-quadruplex (G4) ligands pyridostatin (PDS) and PhenDC3.

**Figure 4:**
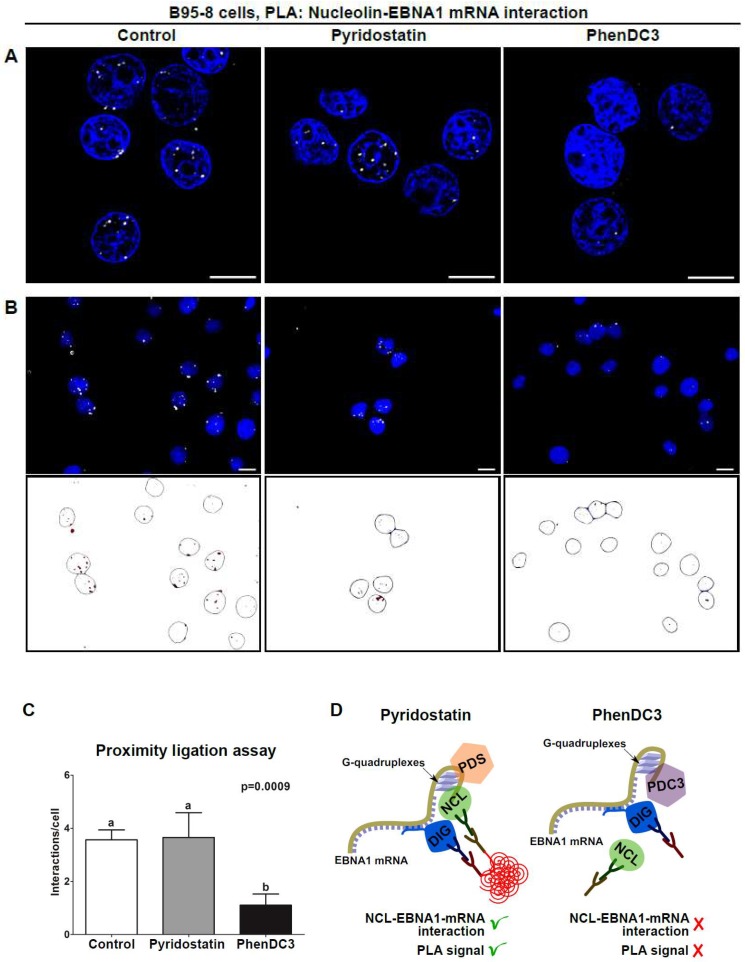
PLA as a tool for the characterization of RNA-binding drugs. (**A**) B95-8 cells treated with the G-quadruplex (G4) ligands pyridostatin (PDS) or PhenDC3 were tested for the NCL-*EBNA1* mRNA interaction using PLA. PhenDC3, but not PDS, was shown to prevent the interaction, denoting that PhenDC3 competes specifically with NCL for binding *EBNA1* mRNA G4. (**B**) Quantitative PLA was performed to quantify the effect of PDS and PhenDC3 on NCL-*EBNA1* mRNA interactions. One hundred cells from control (DMSO)-, PDS-, and PhenDC3-treated groups were imaged and the number of interactions per cell was estimated using a customized protocol in ImageJ. Upper and lower lanes depict images before and after analysis, respectively. Open circles represent the nucleus included in the analysis and red dots depict PLA complexes. Cells located in the border of images and dots found outside filtered nucleus were excluded from the analysis. (**C**) Histogram displaying the number of NCL-*EBNA1* mRNA interactions per cell from control (DMSO)-, PDS-, and PhenDC3-treated groups. Data shown are mean ± SD of three independent experiments performed as described in (**B**). (**D**) Schematic depicting the use of PLA to evaluate the effect of PDS and PhenDC3 on the NCL-*EBNA1* mRNA interactions in EBV-infected B95-8 cells. PhenDC3, but not PDS, competes with NCL for binding *EBNA1* mRNA G4. One possible explanation for this difference (differential binding sites for PhenDC3 and PDS, the latter forming a ternary complex EBNA1 mRNA/NCL/PDS, whereas PhenDC3 and NCL would share a common binding site) is shown. By preventing this interaction, PhenDC3 stimulates the translation of the *EBNA1* mRNA and the production of EBNA1-derived antigenic peptides for the MHC class I pathway. PDS and PDC3 mean pyridostatin and PhenDC3, respectively. Scale bars represent 10 µm.

An estimated 2–3% of cancers are associated with EBV, making GAr-based EBNA1 immune evasion an important target for therapeutic approaches against EBV-related cancers [20,21]. Therefore, the binding of NCL to G4 in *EBNA1* mRNA represents a promising target for novel therapeutic strategies aimed at stimulating immune recognition of EBV-carrying cancers. This, and the fact that different compounds bind the G4 of the *EBNA1* mRNA, makes this an interesting model system for the identification of compounds that interfere with a specific RNA-protein interaction. Since NCL binds specifically G4 within EBNA mRNA [11], we treated, or not, B95.8 cells with the G4 ligands PDS or PhenDC3 (Figure 3) and evaluated the effect of treatments on this interaction using PLA. Of note, PhenDC3, but not PDS, reduced the NCL-*EBNA1* mRNA interaction (Figure 4A). This is surprising as both compounds bind strongly the *EBNA1* mRNA G4 structure in vitro [11,22]. In addition, a previous report has shown that PDS suppresses EBNA1 synthesis in vitro [15], while PhenDC3 increases the levels of EBNA1 in cellulo [11]. Altogether, these contrasting results indicate that different G4 ligands may induce distinct biological responses and complementary assays are required to determine the activity of these compounds in the cellular context. To refine this finding, we developed a protocol to quantify PLA results using the public domain image processing program ImageJ (Figure 4B and Supplementary materials). As advantages, quantitative PLA generates information suitable for statistical tests, avoids subjective data interpretation, and enables the estimation of differences less evident by the comparison of single images. This approach revealed a remarkable difference between the ability of PDS and PhenDC3 in preventing the NCL-*EBNA1* mRNA interaction (Figure 4C). It has been reported that by binding to an RNA fold, small molecules can alter RNA structure, inhibiting or enhancing protein-RNA associations [23]. Therefore, one could speculate that PDS and PhenDC3 may modify G4 structure in a different way, thereby preventing, or not, the binding of NCL, which can help to explain the discrepancy between these two G4 ligands. Alternatively, the difference between these two compounds in their ability to prevent NCL binding to G4 of *EBNA1* mRNA may be due to differential affinities for these G4. In line with this hypothesis, we have previously observed that PDS binding affinity for EBNA1 G4 is significantly weaker when compared to PhenDC3 as evaluated by G4-FID assay: DC_50_ (PDS) ≈ 0.47 µM); DC_50_ (PhenDC3) ≈ 0.26 µM. This was further confirmed by the thermal stabilization values (ΔTm) measured by the FRET-melting assay on EBNA1 G4 (ΔTm PDS = 14.3 °C; ΔTm PhenDC3 = 20.5 °C [11]. Although the two assays are calibrated for selecting high affinity binders, the difference is nonetheless significant. Thus, it is tempting to deduce that the higher affinity of PhenDC3 for the EBNA1 G4 might give it an advantage when placed in a complex cellular milieu, thereby allowing this compound, and not PDS, to prevent NCL binding by a competitive mechanism (Figure 4D). In support of this we have previously shown that PhenDC3 is able to inhibit the pulldown of NCL by EBNA1 G4-coated beads, whereas PDS has no effect in the same conditions [11]. However, one can consider this last possibility quite unlikely, as the affinity of PDS for the *EBNA1* mRNA G4 structure is quite high per se, at least in vitro. Hence, PDS should also prevent NCL binding, although less efficiently than PhenDC3. Finally, another possibility is that PDS and PhenDC3 may have different binding sites on *EBNA1* mRNA G4. In particular, a ternary interaction could exist between PDS/NCL/*EBNA1* mRNA G4 as has been observed in the case of TERRA G4, the G4 ligand carboxypyridostatin (cPDS, a chemical derivative of PDS), and anti-G4 antibodies [24], whereas PhenDC3 and NCL may share the same, or overlapping, binding site(s). The number of binding sites may also strongly differ from one G4 ligand to another, especially with long G4-forming domains like the GAr-encoding sequence of *EBNA1* mRNA [25]. All these possibilities highlight the importance of methods enabling the study of RNA-protein interactions and the control of these interactions by candidate molecules in presence of the multiple biological entities within the cell.

## 4. Concluding Remarks

Protein-mRNA interactions influence multiple aspects of cellular function. However, in spite of progress experienced over the last decades, the study of these interactions has been restricted to assays technically demanding and unable to provide accurate information at subcellular levels. In this context, PLA represents an attractive alternative to overcome these limitations, opening new avenues for the study of protein-mRNA interactions in cellulo. We provided evidences that PLA can be coupled to quantitative analysis and be successfully employed to screen molecules interfering with target protein-mRNA interactions. Additionally, the approach described here can be adapted to other technologies, like flow cytometry, for medium to high throughput drug screening. This highlights the potential of mRNA-protein PLA as a tool for efforts focused not only on targeting specific G4 structures, but more generally for drug development based on disruption of specific protein-RNA structure complexes.

## Supplementary Materials

The supplementary materials are available online.
