# Supplementary material for: In Cellulo Protein-mRNA Interaction Assay to Determine the Action of G-Quadruplex-Binding Molecules
Source: Molecules. 2018 Nov 29;23(12):3124. doi: 10.3390/molecules23123124 (PMC6321085; doi:10.3390/molecules23123124)

Quantitative proximity ligation assay – analysis of images with ImageJ

This customized protocol enables the quantification of interactions detected by the proximity ligation assay (PLA) using the public domain image processing program ImageJ. The image below will be used as example.

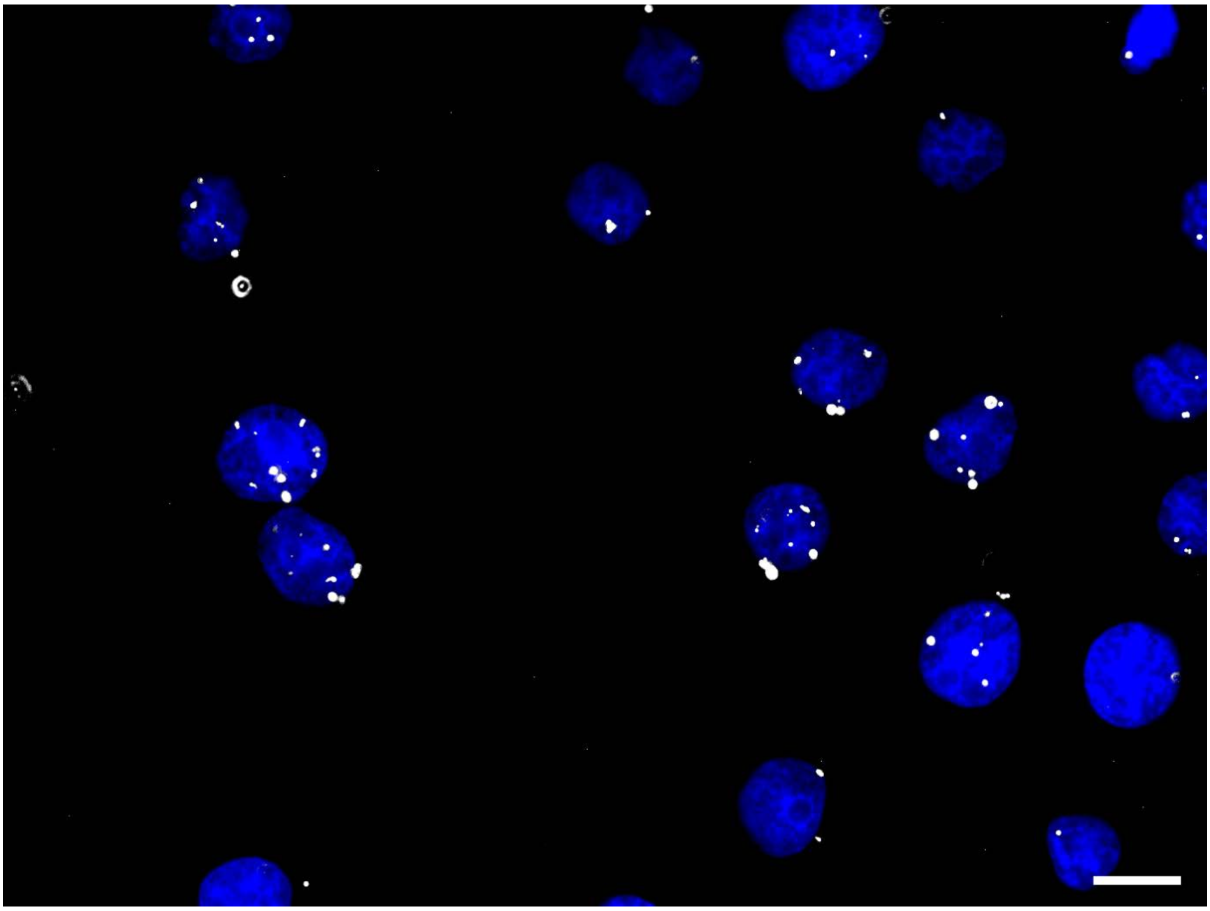

1 – Split multi-color images into single channels (Image → Color → Split Channels)

2 – Check that the "scale when converting" option is selected (Edit → Options → Conversions → Scale when converting)

3 – Convert single channel images to grayscale (Image → Type → 16 bit)

4 – Create a binary image and adjust threshold to highlight the structures of interest (Image → Adjust → Threshold). The structures of interest will be highlighted in red. After subtracting background pixels, click "apply". This creates a binary image with only two pixel intensities: black = 0 and white = 255 (image below). Since this protocol was optimized for quantifying nuclear interactions, in the first round of analysis, threshold is adjusted to select DAPI-stained

nuclei. However, cytoplasmic or other stainings can be employed to select other cell components.

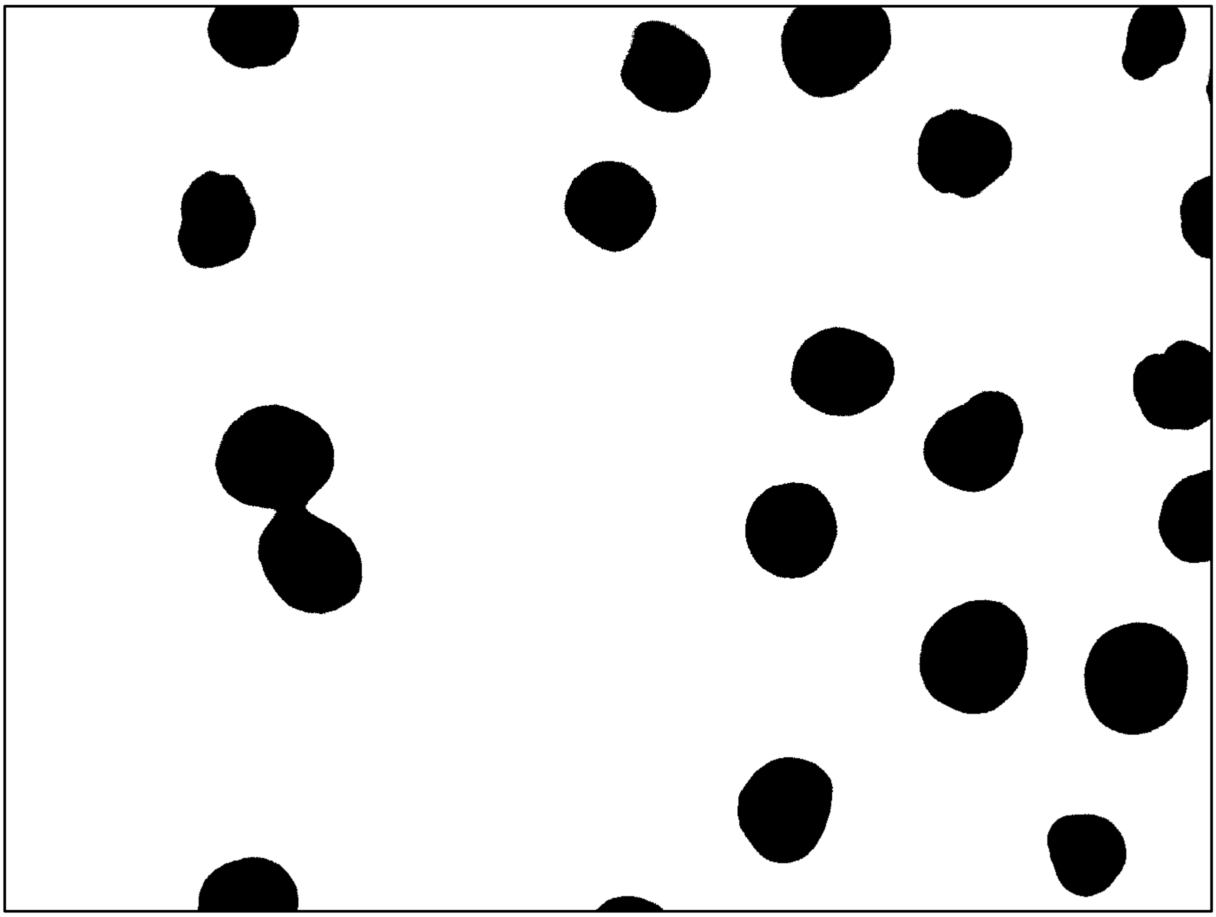

5 – Split nuclei merged together (Process → Binary → Watershed)

6 – Adjust parameters for analysis. This protocol does not take into account the area or other pixels data from selected structures. Therefore, just click on “display label” to label your data table with the image name and structure number. In “Redirect to”, select the binary image to be analyzed.

7 – Analyze the selected image (Analyze → Analyze Particles). For counting nuclei, in “Size”, use “0.03-infinity”. This parameter must be adjusted according to the cell line employed or the structure of interest. For analyzing PLA complexes, adjust size as “0.0003-infinity”. In “Show”, select “outlines” to generate an image of selected structures. Select the “Display results”, “clear results” and “Summarize” options. The “Exclude on the edges” box must be selected to avoid cells that are not entirely visualized in the image. An image of nuclei included in the analysis is generated and the number of selected nuclei is described in a summary table (below)

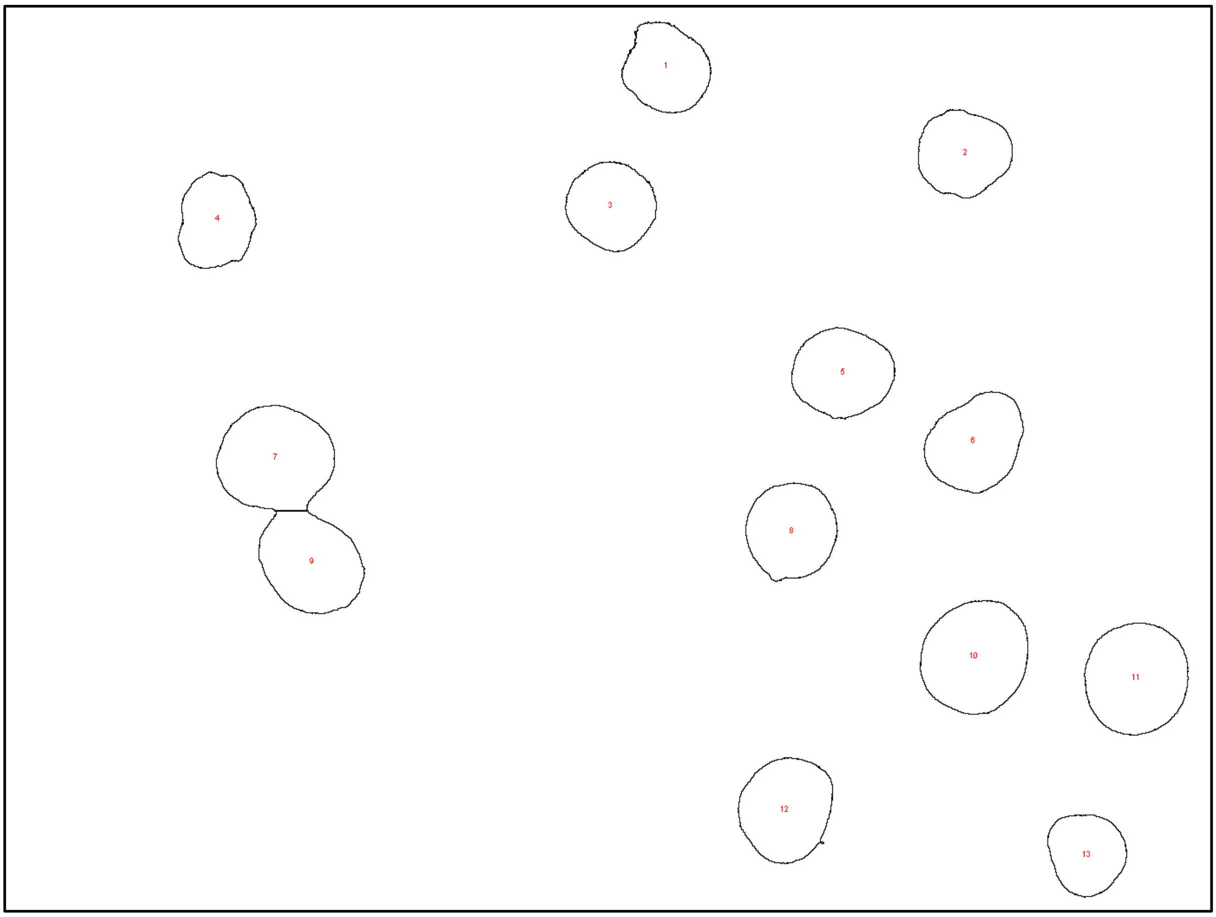

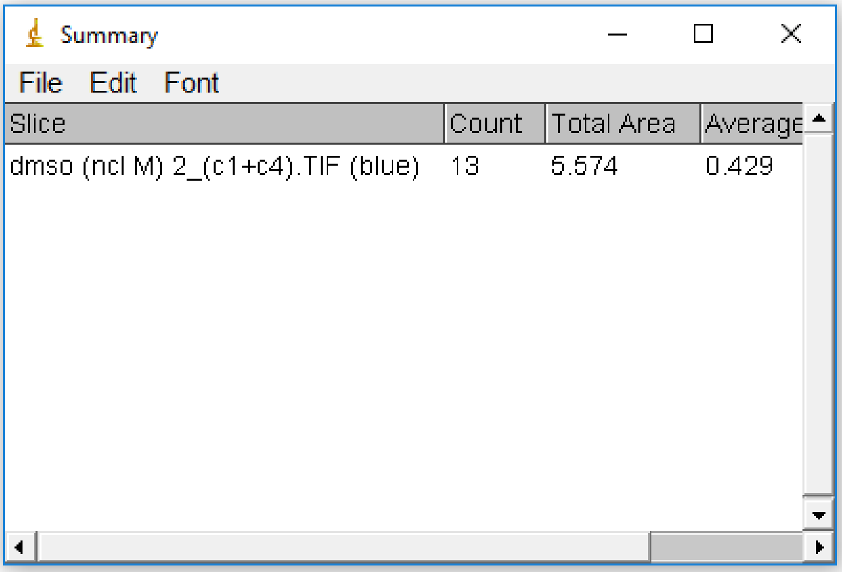

8 – Sava data from the first round of analysis and keep the image of nuclear outlines

9 – Repeat steps 4 – 7 to analyze the image corresponding to PLA complexes. This will generate a new summary table and outline image (below)

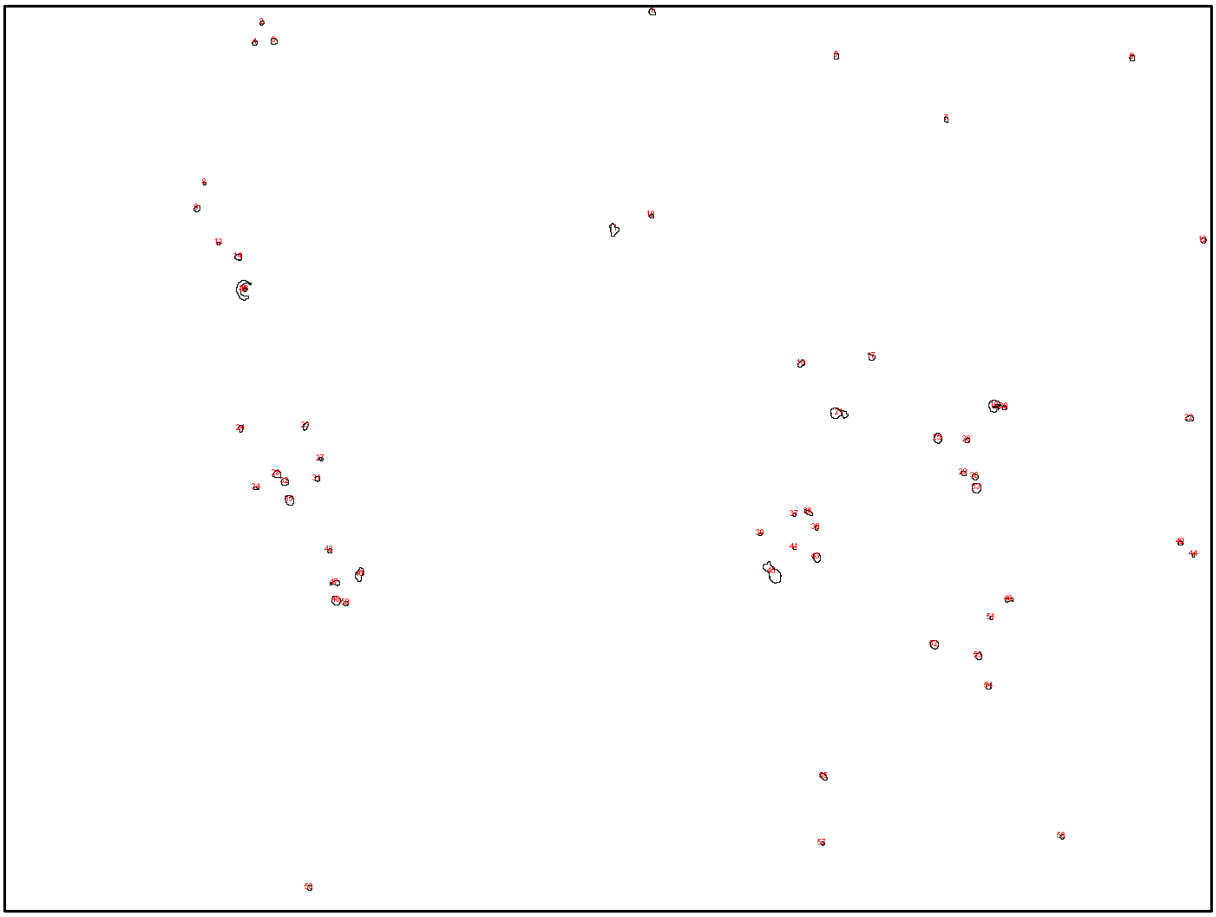

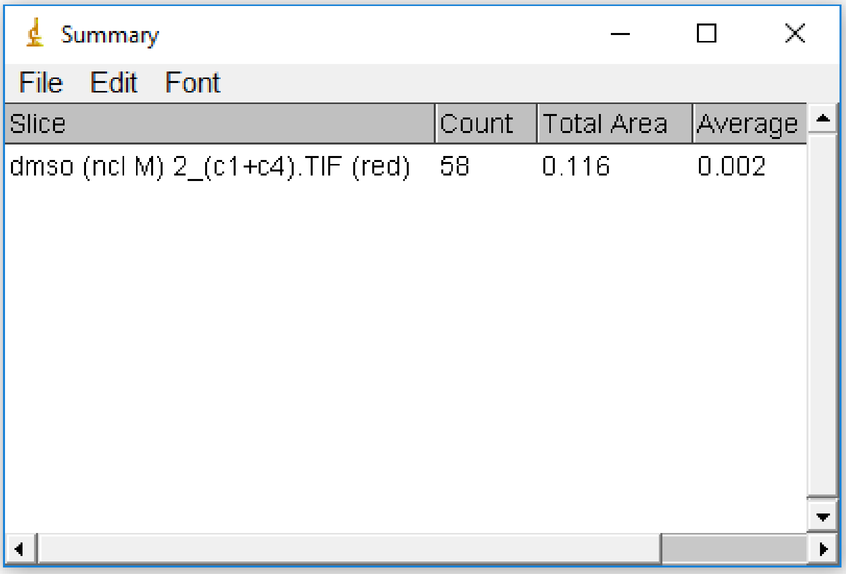

10 – Invert outline images corresponding to nuclei and PLA complexes (edit → invert)

11 – Merge inverted images corresponding to nuclei and PLA complexes (image → color → merge). Select nuclei image as “blue” and PLA image as “red”. This will generate an image

displaying selected nuclei and PLA complexes in blue and red, respectively (below). Exclude PLA complexes outside selected nuclei and calculate the average number of complexes per cell.

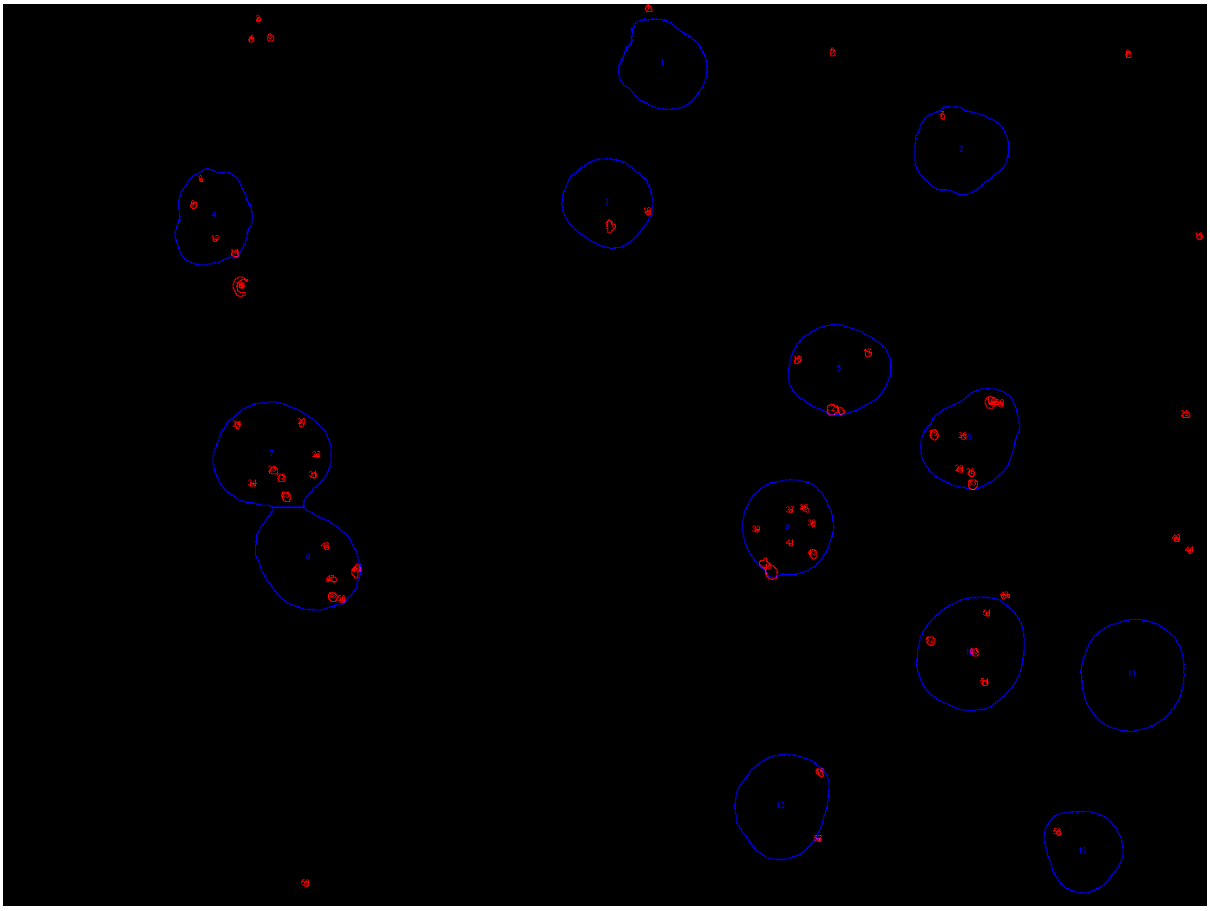

Supplement: Supplementary file 1 [file molecules-23-03124-s001.pdf]
